# Delamanid Added to an Optimized Background Regimen in Children with Multidrug-Resistant Tuberculosis: Results of a Phase I/II Clinical Trial

**DOI:** 10.1128/aac.02144-21

**Published:** 2022-04-11

**Authors:** Anthony J. Garcia-Prats, Melchior Frias, Louvina van der Laan, Anjanette De Leon, Maria Tarcela Gler, H. Simon Schaaf, Anneke C. Hesseling, Suresh Malikaarjun, Jeffrey Hafkin

**Affiliations:** a Desmond Tutu TB Centre, Department of Paediatrics and Child Health, Faculty of Medicine and Health Sciences, Stellenbosch University, Cape Town, South Africa; b De La Salle Health Science Center, Cavite, Philippines; c Lung Center of the Philippines, Quezon City, Philippines; d Otsuka Pharmaceutical Development & Commercialization, Rockville, Maryland, USA

**Keywords:** delamanid, pediatric tuberculosis, multidrug-resistant tuberculosis, safety, efficacy, pharmacokinetics, age deescalation study

## Abstract

Delamanid has been demonstrated to be safe and effective for treatment of adult multidrug-resistant tuberculosis (MDR-TB) and has been approved by the European Commission for treatment of pediatric MDR-TB patients at least 10 kg in weight, making the drug no longer limited to adults. A 10-day phase I age deescalation study was conducted, followed by a 6-month phase II extension study, to assess the pharmacokinetics, safety, tolerability, and preliminary efficacy of delamanid when combined with optimized background regimen (OBR) in children (birth to 17 years) with MDR-TB. Delamanid administered at 100 mg twice-daily (BID), 50 mg BID, and 25 mg BID resulted in exposures in 12- to 17- (*n* = 7), 6- to 11- (*n* = 6), and 3- to 5-year-olds (*n* = 12), respectively, comparable with those in adults at the approved adult dosage (100 mg BID). Exposures in 0- to 2-year-olds (*n* = 12) following a weight-based dosing regimen (5 mg once daily [QD] to 10 mg BID) were lower than predicted from pharmacokinetic modeling of the older three age groups and below target exposures in adults. Overall, the safety profile of delamanid in children 0 to 17 years of age was similar to the adult profile. At 24 months after the first delamanid dose, 33/37 children (89.2%) had favorable treatment outcomes, as defined by the World Health Organization (15/37 [40.5%] cured and 18/37 [48.6%] completed treatment). A new pediatric delamanid formulation used in 0- to 2-year-olds and 3- to 5-year-olds was palatable per child/parent and nurse/investigator reports. Data from initial phase I/II studies inform our understanding of delamanid use in children and support its further assessment in the setting of pediatric MDR-TB. (This study has been registered at ClinicalTrials.gov under identifiers NCT01856634 [phase I trial] and NCT01859923 [phase II trial].).

## INTRODUCTION

Worldwide, 25,000 to 32,000 children develop multidrug-resistant tuberculosis (MDR-TB; Mycobacterium tuberculosis resistant to isoniazid and rifampin) every year (1, 2). It is estimated that only 3 to 4% of these children receive appropriate treatment and that their overall mortality rate is 21% (3). Among those who do receive appropriate therapy, the majority have good outcomes. Nonetheless, in a meta-analysis of 975 children with MDR-TB, most of whom had received local standard of care informed by international recommendations, 10% either died or failed treatment (4). The persistence of high treatment-failure rates, combined with the long duration of treatment for MDR-TB, high rates of toxicity associated with older second-line drugs (5, 6), and absence of palatable child-friendly formulations, demonstrates the need for new pediatric MDR-TB drugs and regimens.

Delamanid (Deltyba, Otsuka Pharmaceutical Co, Ltd., Tokyo, Japan) is a bicyclic nitroimidazooxazole compound that inhibits the synthesis of mycolic acids (7), key components of the lipid-rich cell wall of M. tuberculosis (8). In preclinical studies, delamanid exhibited the lowest MIC among approved TB drugs against drug-susceptible and drug-resistant isolates of M. tuberculosis (9). Delamanid pharmacokinetics in adults with MDR-TB was characterized by first-order elimination and absorption, an absorption lag time, and decreased relative bioavailability with increasing dose (10). At steady state, delamanid 100 mg twice-daily (BID) resulted in a mean peak serum concentration (*C*_max_) of 414 ng/mL, a mean area under the 24-h concentration-time curve (AUC_0–24 h_) of 7,925 h × ng/mL, and an elimination half-life of 30 to 38 h, with albumin appearing to be predominantly responsible for metabolizing delamanid to its primary metabolite (11, 12). No clinically relevant drug-drug interactions with antiretroviral drugs or anti-TB drugs were observed in healthy individuals (13), but absorption of delamanid increased significantly when taken with food (14).

In adults with MDR-TB, delamanid plus an optimized background regimen (OBR) resulted in significantly higher sputum culture conversion rates than placebo plus OBR after 2 months of therapy (45.4% versus 29.6%; *P* = 0.0083) (11). Overall, the safety profile of delamanid was favorable, with the most common adverse events being gastrointestinal upset (nausea, vomiting, and upper abdominal pain), insomnia, and headache. Patients in the delamanid plus OBR group had more episodes of QT-interval prolongation, although none were associated with clinical manifestations such as syncope or arrhythmias. In another randomized, placebo-controlled study in adults with MDR-TB, median time to sputum culture conversion was lower in the delamanid plus OBR arm than in the placebo plus OBR arm, but the difference did not reach statistical significance (*P* = 0.0562 for the comparison) (15). Delamanid has been approved in 14 countries as part of an appropriate combination regimen for pulmonary MDR-TB in adults when an effective treatment regimen cannot otherwise be composed for reasons of resistance or tolerability (16). The currently recommended adult dosage is 100 mg twice-daily (BID).

Preliminary case series have indicated that delamanid may also have utility in children (17), and recently the drug has been approved in the EU at doses of 100 mg BID and 50 mg BID for adolescents and children with MDR-TB whose body weights are ≥50 kg and ≥30 kg to <50 kg, respectively (16). The current report describes the initial clinical studies of a pediatric developmental program aimed at characterizing the pharmacokinetics, safety, tolerability, and efficacy of delamanid in children with MDR-TB.

## RESULTS

### Study population.

A total of 44 children were screened for the phase I study, 37 of whom enrolled in the trial: 7 in group 1 (12 to 17 years), 6 in group 2 (6 to 11 years), 12 in group 3 (3 to 5 years), and 12 in group 4 (0 to 2 years) (Fig. S1). All enrolled children completed the phase I study and enrolled in the phase II study. Thirty-five (94.6%) in the phase II extension completed the trial, i.e., were evaluated 24 months after the first phase II dose of delamanid. The remaining two patients, 1 in group 3 and 1 in group 4, died from new-onset (non-TB) pneumonia before reaching the end of the trial (both deaths were assessed as unrelated to delamanid plus OBR). Demographic and clinical features at baseline for the phase I study population are summarized in Table 1. Details of optimized background regimens are summarized in Table S1.

**TABLE 1:** Demographics and baseline characteristics: phase I population of children with multidrug-resistant tuberculosis receiving delamanid and an optimized background regimen

Characteristic	Value for group:				
	12–17 yrs (*n* = 7)	6–11 yrs (*n* = 6)	3–5 yrs (*n* = 12)	0–2 yrs (*n* = 12)	Total (*n* = 37)
Age, median yrs (range)	15.50 (13.3–17.5)	9.55 (7.3–11.4)	4.35 (3.1–5.9)	1.65 (0.7–2.5)	4.40 (0.7–17.5)
Male, *n* (%)	4 (57.1)	2 (33.3)	6 (50.0)	6 (50.0)	18 (48.6)
Wt, median kg (range)	38.5 (34–45)	25.0 (16–34)	14.2 (10–19)	10.1 (6–13)	14.3 (6–45)
Race, *n* (%)					
Asian	7 (100.0)	4 (66.7)	8 (66.7)	6 (50.0)	25 (67.6)
Black	0 (0.0)	0 (0.0)	2 (16.7)	0 (0.0)	2 (5.4)
Mixed or other	0 (0.0)	2 (33.3)	2 (16.7)	6 (50.0)	10 (27.0)
MDR-TB diagnostic criteria, *n* (%)					
Confirmed^*a*^	6 (86)	3 (50)	3 (25)	3 (25)	15 (41)
Presumptive^*b*^	1 (14)	3 (50)	9 (75)	9 (75)	22 (59)
Chest radiograph, *n* (%)					
Not done	0 (0.0)	0 (0.0)	1 (8.3)	0 (0.0)	1 (2.7)
Normal	0 (0.0)	1 (16.7)	0 (0.0)	0 (0.0)	1 (2.7)
Abnormal	7 (100.0)	5 (83.3)	11 (91.7)	12 (100.0)	35 (94.6)
Findings on chest radiograph at time of diagnosis consistent with TB, *n* (%)	7 (100.0)	4 (66.7)	11 (91.7)	10 (83.3)	32 (86.5)
Site of disease, *n* (%)					
Pulmonary only	7 (100.0)	2 (33.3)	7 (58.3)	10 (83.3)	26 (70.3)
Extrapulmonary only	0	1 (16.7)	1 (8.3)	0 (0.0)	2 (5.4)
Both pulmonary and extrapulmonary	0	3 (50.0)	4 (33.3)	2 (16.7)	9 (24.3)

a Confirmed diagnosis: a child with laboratory diagnosis of MDR-TB (i.e., culture with a drug susceptibility test and/or rapid testing).

b Presumptive diagnosis: a child with TB and a recent close contact with MDR-TB, a child who fails to improve while adherent to first-line anti-TB treatment, or a child with an adult source case who is a treatment failure, a retreatment case, or recently died from TB.

### Pharmacokinetics.

**FIG 1:**
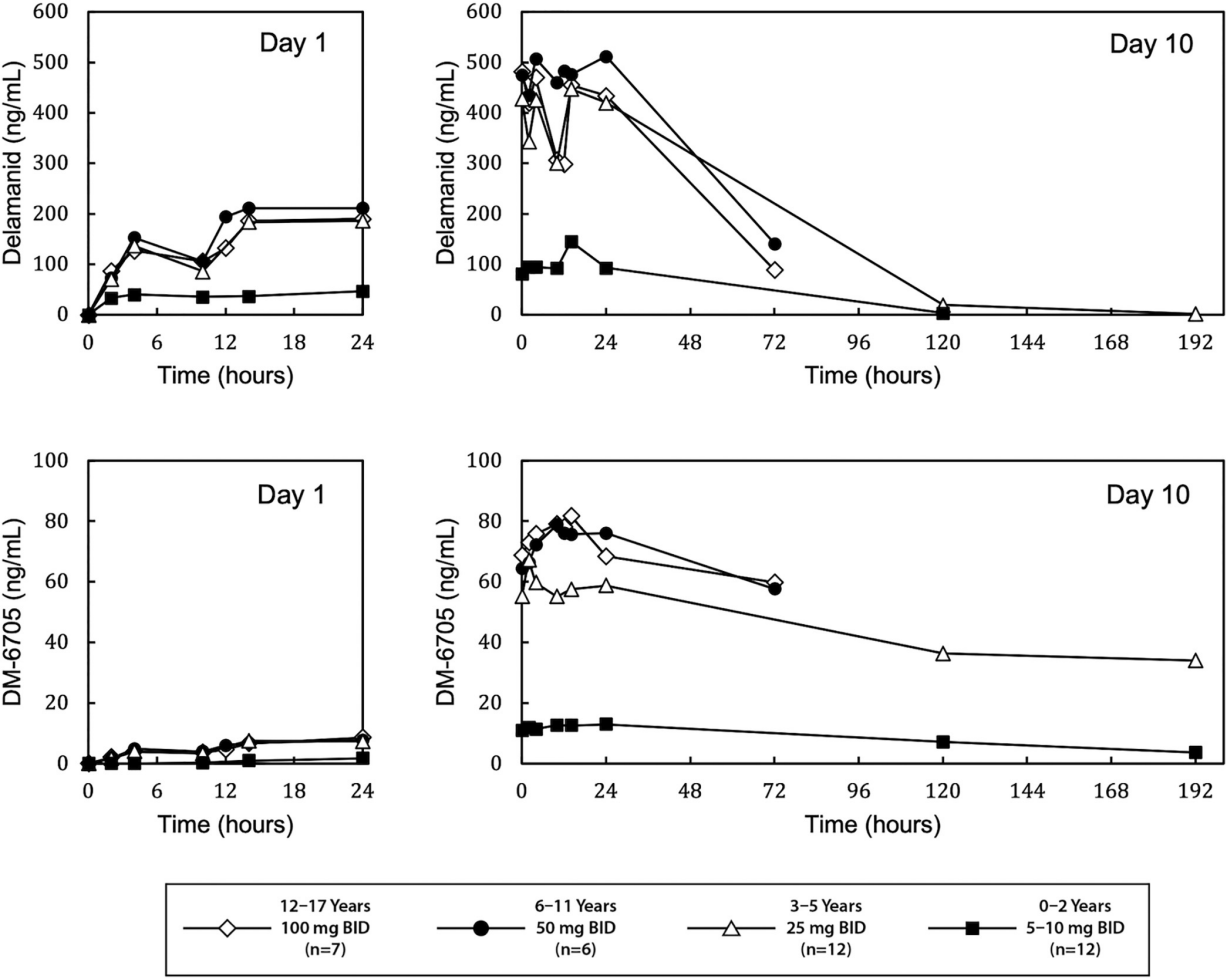
Median plasma concentration versus time profiles for delamanid (top) and DM-6705 (bottom) following oral doses of delamanid on day 1 and day 10 in pediatric patients with multidrug-resistant tuberculosis. Delamanid was administered in combination with an optimized background regimen.

At steady state (day 10), groups 1, 2, and 3 had similar peak delamanid plasma concentrations (median *C*_max_: 557, 573, and 500 ng/mL, respectively) and delamanid exposures (median AUC_0–24 h_: 9,790, 12,000, and 9,290 ng × hr/mL, respectively) (Fig. 1 and Table 2). Group 4 had lower median steady-state values for *C*_max_ (179 ng/mL) and AUC_0–24 h_ (2,740 ng × hr/mL). Delamanid oral clearances were 341, 139, 89.8, and 87.4 mL/min in groups 1, 2, 3, and 4, respectively. Across the four groups, delamanid exposure increased consistently by 2.68-, 2.55-, 2.70-, and 2.69-fold, respectively, at day 10 compared to day 1.

**TABLE 2:** Pharmacokinetic parameters for delamanid and the delamanid metabolite DM-6705 following oral dosing on day 10 in children with multidrug-resistant tuberculosis^*a*^^,^^*b*^

Parameter	Value for group:				
	12–17 yrs (*n* = 7)^*c*^	6–11 yrs (*n* = 6)^*c*^	3–5 yrs (*n* = 12)^*d*^	0–2 yrs (*n* = 12)^*e*^	Adults (*n* = 744)^*f*^
Delamanid dose	100 mg BID	50 mg BID	25 mg BID	10 mg BID, 5 mg BID, or 5 mg QD	100 mg BID
Delamanid PK parameter					
*C*_max_ (ng/mL)	557 (304–803)	573 (485–682)	500 (287–919)	179 (45.2–298)	333 (28)^*g*^
*t*_max_ (h)	3.98 (0.0–24.0)	11.98 (2.0–24.0)	4.00 (0.0–24.0)	13.75 (2.0–23.97)	
AUC_0–24 h_ (ng × h/mL)	9,790 (6,170–13,000)	12,000 (9,810–13,300)	9,290 (5,180–12,900)	2,740 (701–4,910)	6,863 (31)^*g*^
CL/F (mL/min)	341 (257–541)	139 (125–170)	89.8 (64.5–161)	87.4 (67.9–123)	655 (625–685)^*h*^
t_1/2, z_ (h)	30.4 (19.7–54.7)	23.7 (19.1–82.1)	20.5 (16.8–31.3)	ND	30–38^*i*^
R_ac_AUC_0–24 h_	2.68 (1.86–4.71)	2.55 (1.72–4.11)	2.70 (1.85–4.00)	2.69 (1.24–4.20)	3.0–3.3^*h*^
DM-6705 PK parameter					
*C*_max_ (ng/mL)	81.7 (52.9–93.2)	90.0 (62.4–112)	68.7 (33.7–95.0)	14.2 (2.38–35.9)	
*t*_max_ (h)	12.0 (10.0–14.0)	12.0 (2.0–24.0)	3·00 (0.0–24.0)	10.49 (1.97–24.0)	
AUC_0–24 h_ (ng × h/mL)	1,780 (1,210–2,010)	1,880 (1,210–2,210)	1,370 (671–2,160)	291 (49.6–774)	
*t*_1/2, z_ (h)	237.3 (217.0–350.5)	ND	155.4 (88.8–341.8)	128.2 (70.0–201.4)	
R_ac_AUC_0–24 h_	12.90 (7.87–19.95)	11.0 (6.3–23.44)	11.15 (6.15–19.83)	15.19 (5.23–65.26)	
Ratio of delamanid/DM-6705 AUC_0–24 h_	0.18 (0.155–0.198)	0.148 (0.123–0.181)	0.164 (0.119–0.190)	0.12 (0.071–0.161)	

a AUC_0–24 h_, area under the plasma-time concentration curve from time 0 to 24 h; BID, twice-daily; *C*_max_, peak (maximum) concentration of drug in plasma; CL/F, oral clearance; ND, not determined; R_ac_, accumulation ratio; *t*_1/2, z_, terminal phase elimination half-life; *t*_max_, time of peak concentration (*C*_max_).

b Delamanid was administered in combination with an optimized background regimen. All values are expressed as median (range). Included for comparison are published PK data from adults with multidrug-resistant TB receiving delamanid (10).

c Group 1 and group 2 received adult formulation delamanid (2× 50 mg tablet and 1× 50 mg tablet, respectively).

d Group 3 received delamanid pediatric formulation (1× 25 mg dispersible tablet).

e Group 4 received delamanid pediatric formulation (administered as 5 mg dispersible tablets) according to their baseline body weights, i.e., patients who were >10 kg received 10 mg BID, >8 and ≤10 kg received 5 mg BID, and ≥5.5 kg and ≤8 kg received 5 mg QD.

f From Wang et al. (10).

g Predicted steady-state parameters in a non-Asian male (55 kg) with serum albumin of >3.4 g/dL. Shown are means (coefficient of variation percentage [CV%]).

h In a 55 kg male with serum albumin of >3.4 g/dL. For CL/F, shown are means (95% confidence interval [CI]).

i From Gler et al. (11).

For DM-6705, the primary metabolite of delamanid, groups 1, 2, and 3 again had approximately similar steady-state median values for AUC_0–24 h_ (1,780, 1,880, and 1,370 ng × hr/mL, respectively) and *C*_max_ (81.7, 90.0, and 68.7 ng/mL, respectively), while group 4 had lower median values for the same parameters (291 ng × hr/mL and 14.2 ng/mL) (Fig. 1 and Table 2). The ratios of delamanid exposure to DM-6705 exposure at steady state were 0.18, 0.15, 0.16, and 0.12 in groups 1 through 4, respectively. Pharmacokinetic parameters for delamanid and DM-6705 on day 1 are provided in Table S2.

### Safety.

In the phase I population (*n* = 37), the most common treatment-emergent adverse events in children who received delamanid plus OBR were vomiting (*n* = 9 [24.3%]), pyrexia (*n* = 7 [18.9%]), nausea (*n* = 5 [13.5%]), toothache (*n* = 5 [13.5%]), and hyperuricemia (*n* = 5 [13.5%]) (Table 3; a full list of all treatment-emergent adverse events in the phase I safety population is provided in Table S3). Two children (5.4%) experienced serious adverse events (acute hepatitis A infection and lower respiratory tract infection in a 3- to 5-year-old and a 0- to 2-year-old, respectively), both of which were assessed as unrelated to study treatment. Nine children (24.3%) experienced at least one adverse event potentially related to delamanid plus OBR. The most common treatment-related events by System Organ Class were gastrointestinal disorders (*n* = 4 [10.8%]), skin and subcutaneous tissue disorders (*n* = 3 [8.1%]), and investigations (*n* = 3 [8.1%]; includes 2 cases of QT prolongation [both in 3- to 5-year-old group] and 1 case each of PR prolongation [3- to 5-year-old], U wave present [3- to 5-year-old], and prothrombin time prolonged [0- to 2-year-old]). The only treatment-related events that occurred in more than one patient were diarrhea (*n* = 2 [5.4%]) and electrocardiogram (ECG) QT-interval prolongation. All treatment-related adverse events were mild or moderate in severity.

**TABLE 3:** Treatment-emergent adverse events occurring in >10% of total patients on delamanid plus an optimized background regimen

Adverse event, *n* (%)	Value for group:				
	12–17 yrs (*n* = 7)	6–11 yrs (*n* = 6)	3–5 yrs (*n* = 12)	0–2 yrs (*n* = 12)	Total (*n* = 37)
Phase I					
Vomiting	2 (28.6)	2 (33.3)	3 (25.0)	2 (16.7)	9 (24.3)
Pyrexia	2 (28.6)	0 (0.0)	4 (33.3)	1 (8.3)	7 (18.9)
Nausea	4 (57.1)	0 (0.0)	1 (8.3)	0 (0.0)	5 (13.5)
Toothache	1 (14.3)	2 (33.3)	2 (16.7)	0 (0.0)	5 (13.5)
Hyperuricemia	2 (28.6)	1 (16.7)	0 (0.0)	2 (16.7)	5 (13.5)
Lower respiratory tract infection	0 (0.0)	0 (0.0)	3 (25.0)	1 (8.3)	4 (10.8)
Upper respiratory tract infection	0 (0.0)	1 (16.7)	0 (0.0)	3 (25.0)	4 (10.8)
Arthralgia	2 (28.6)	1 (16.7)	1 (8.3)	0 (0.0)	4 (10.8)
Headache	2 (28.6)	1 (16.7)	1 (8.3)	0 (0.0)	4 (10.8)
Phase II extension					
Upper respiratory tract infection	4 (57.1)	3 (50.0)	5 (41.7)	2 (16.7)	14 (37.8)
Headache	5 (71.4)	3 (50.0)	2 (16.7)	0 (0.0)	10 (27.0)
Hyperuricemia	2 (28.6)	1 (16.7)	4 (33.3)	3 (25.0)	10 (27.0)
Arthralgia	3 (42.9)	2 (33.3)	3 (25.0)	0 (0.0)	8 (21.6)
Gastroenteritis	1 (14.3)	1 (16.7)	1 (8.3)	4 (33.3)	7 (18.9)
Pneumonia	1 (14.3)	1 (16.7)	3 (25.0)	2 (16.7)	7 (18.9)
Vomiting	2 (28.6)	1 (16.7)	2 (16.7)	1 (8.3)	6 (16.2)
Lower respiratory tract infection	0 (0.0)	1 (16.7)	3 (25.0)	2 (16.7)	6 (16.2)
Hypothyroidism	0 (0.0)	1 (16.7)	2 (16.7)	2 (16.7)	5 (13.5)
Pyrexia	2 (28.6)	0 (0.0)	2 (16.7)	1 (8.3)	5 (13.5)
Respiratory tract infection	0 (0.0)	0 (0.0)	1 (8.3)	4 (33.3)	5 (13.5)
Otitis media	0 (0.0)	0 (0.0)	1 (8.3)	3 (25.0)	4 (10.8)
Skin laceration	0 (0.0)	1 (16.7)	2 (16.7)	1 (8.3)	4 (10.8)
Wt decreased	0 (0.0)	0 (0.0)	1 (8.3)	3 (25.0)	4 (10.8)

In the phase II population (*n* = 37), the most common events in children who received delamanid plus OBR were upper respiratory tract infection (*n* = 14 [37.8%]), headache (*n* = 10 [27.0%]), hyperuricemia (*n* = 10 [27.0%]), and arthralgia (*n* = 8 [21.6%]) (Table 3; a full list of all treatment-emergent adverse events in the phase II safety population is provided in Table S4). Eight children (21.6%) experienced serious adverse events, two of which were assessed as potentially related to delamanid plus OBR: one case of immune thrombocytopenia and one case of bronchial hyperreactivity, both in group 4. In total, 9 children (24.3%) experienced at least one adverse event that was potentially related to study treatment. The most common treatment-related events were prothrombin time prolonged (*n* = 3 [8.1%]), blood corticotrophin increased (*n* = 2 [5.4%]), liver function test increased (*n* = 2 [5.4%]), and butterfly rash (*n* = 2 [5.4%]). With the exception of the immune thrombocytopenia and bronchial hyperreactivity events described above, which were severe in intensity, all other potentially treatment-related adverse events in the phase II trial were mild to moderate in intensity.

ECG abnormalities observed during treatment are presented in Table S5 and S6. In both studies, new-onset QT interval correction using Fridercia's formula (QTcF) of ≥480 ms was not observed in any age group. In the phase I study, new-onset QTcF of >450 ms occurred in 1 (14.2%) and 2 (16.6%) patients in the 12- to 17-year-old and 3-old to 5-year-old age groups, respectively. In the phase II study, new-onset QTcF of >450 ms occurred in 3 (42.9%) and 2 (33.3%) patients in the 12- to 17-year-old and 6- to 11-year-old age groups, respectively. No patients in the phase I study and 2 (5.6%) patients in the phase II study (1 [14.3%] 12- to 17-year-old and 1 [9.1%] 0- to 2-year-old) had a change from baseline in QTcF interval of >60 ms.

### Palatability.

**FIG 2:**
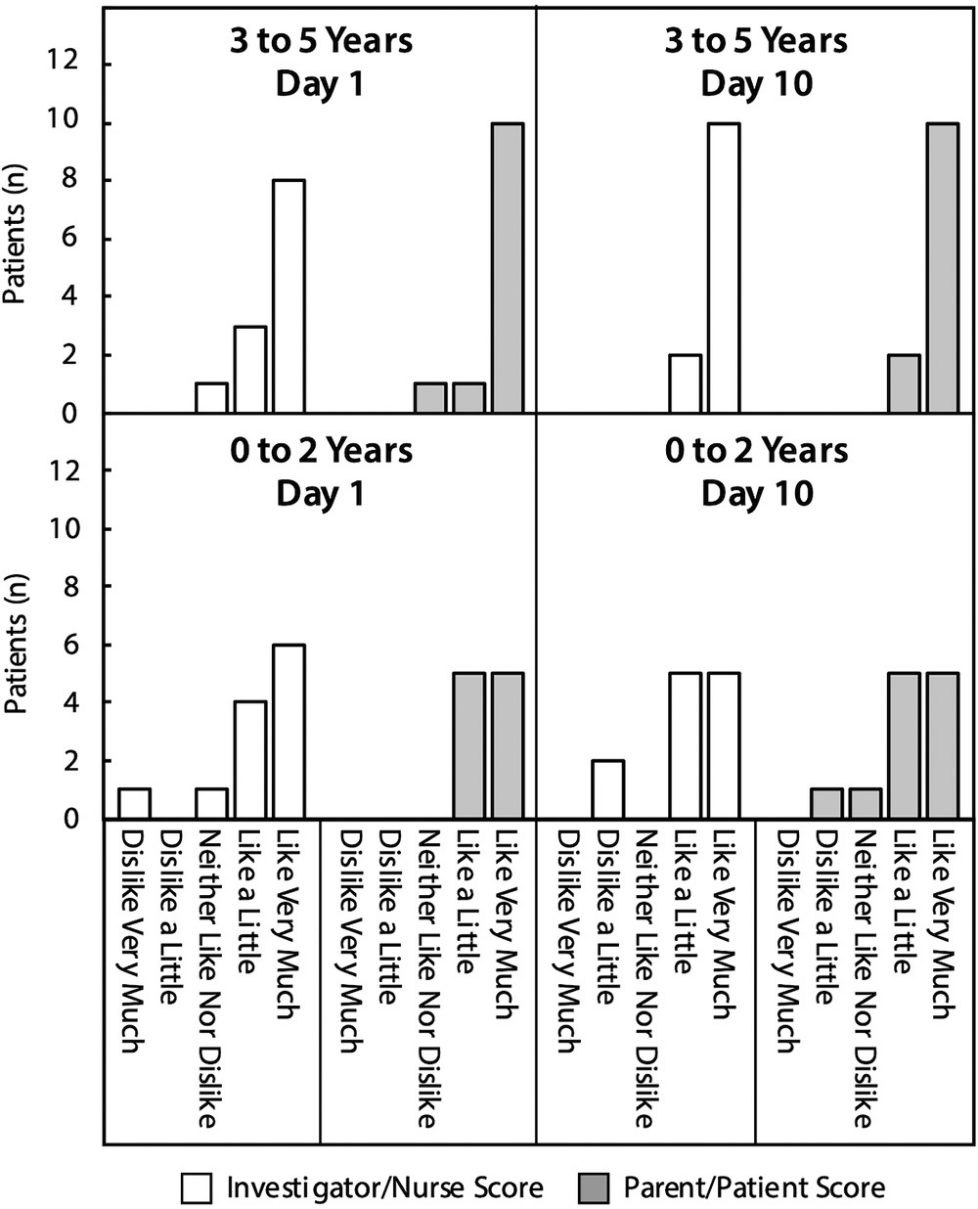
Palatability of the delamanid pediatric formulation in pediatric patients. Palatability assessment categories included “dislike very much,” “dislike a little,” “neither like nor dislike,” “like a little,” and “like very much.”

Palatability of the new pediatric formulation was evaluated in groups 3 and 4. On day 1 of the phase 1 study, 22 child/parent pairs described their first dose of the pediatric formulation as “like very much” (*n* = 15), “like a little” (*n* = 6), or “neither liked nor disliked” (*n* = 1), while none described it as “dislike a little” or “dislike very much” (Fig. 2). On day 10, 23 child/parent pairs described the pediatric formulation as “like very much” (*n* = 15), “like a little” (*n* = 7), or “neither liked nor disliked” (*n* = 1), with only one describing it as “dislike a little.” These acceptable trends were replicated in the investigator/nurse assessments (Fig. 2), as well as in child/parent and investigator/nurse assessments conducted in the phase II study (data not shown).

### Efficacy.

As defined by WHO guidance, 33/37 children (89.2%) had favorable outcomes at 24 months, i.e., 15/37 children (40.5%) were considered cured of MDR-TB and 18/37 (48.6%) achieved treatment completion (Table 4). Of the 4/37 children (10.8%) with unfavorable outcomes at 24 months, 2/37 (5.4%) were lost to follow-up and 2/37 (5.4%) died.

**TABLE 4:** Treatment outcome 24 months after starting therapy with delamanid plus an optimized background regimen in pediatric patients with multidrug-resistant tuberculosis

Outcome, *n* (%)	Value for group:				
	12–17 yrs (*n* = 7)	6–11 yrs (*n* = 6)	3–5 yrs (*n* = 12)	0–2 yrs (*n* = 12)	Total (*n* = 37)
Cured	4 (57.1)	3 (50.0)	3 (25.0)	5 (41.7)	15 (40.5)
Treatment completed^*a*^	2 (28.6)	3 (50.0)	7 (58.3)	6 (50.0)	18 (48.6)
Died	0 (0.0)	0 (0.0)	1 (8.3)^*b*^	1 (8.3)^*c*^	2 (5.4)
Lost to follow-up	1 (14.3)	0 (0.0)	1 (8.3)	0 (0.0)	2 (5.4)

a Per World Health Organization guidance, “treatment completed” patients were those who completed treatment but did not have sufficient microbiologic data to be considered cured.

b The serious adverse events of community acquired pneumonia (death), oral candidiasis, and vaginal candidiasis were assessed as not related to delamanid.

c The serious adverse event of pneumonia (death) was assessed as not related to delamanid.

## DISCUSSION

The current recommended dose of delamanid in adults with pulmonary MDR-TB (100 mg BID) was adopted after extensive pharmacokinetic, safety, and efficacy analyses (10–13, 18, 19). Validation of these empirical results was provided in a recent pharmacokinetic-pharmacodynamic study, which showed that the ratio of AUC to MIC was the primary driver of mycobacterial killing and that delamanid 100 mg BID achieved a cumulative fraction of response of ≥95% in two large trials of adult patients with pulmonary MDR-TB (18). Median delamanid exposures in the current study’s top three age bands—100 mg BID in 12- to 17-year-olds, 50 mg BID in 6- to 11-year-olds, and 25 mg BID in 3- to 5-year-olds—ranged from 9,290 to 12,000 ng × h/mL, similar to or slightly higher than delamanid exposures in adults following 100 mg BID dosing. Therefore, these three pediatric dosing regimens would be expected to provide high levels of mycobacterial killing, assuming the MIC distribution of bacterial populations is similar in infected children and adults. This conclusion confirms guidance from the WHO, which has endorsed the use of delamanid for multidrug- or rifampicin-resistant TB in children aged 3 years or more who are on longer regimens (20), as well as the recent approval of delamanid in the EU for children and adolescents with MDR-TB (16). Population pharmacokinetic modeling with dosing simulations was recently described that can be used to provide more nuanced evidence to inform optimal pediatric delamanid dosing (21).

The weight-based dosing regimens for group 4 (birth to 2 years) in the trial were determined by modeling pharmacokinetic data from the previous three groups. The final steady-state exposure in group 4, however, was significantly lower than anticipated (2,740 ng × h/mL). Since oral bioavailability is a factor of intrinsic clearance and bioavailability, the decreased exposure could be due to either increased clearance or overestimation of bioavailability in the lowest age group. The ratio of delamanid exposure to DM-6705 exposure at steady state ranged from 0.12 to 0.18 in the four groups, which was much lower than the steady-state mean ratio of 0.41 in adults following a regimen of 100 mg BID (internal correspondence). Since delamanid exposures were comparable to those of adults, the lower parent drug-to-metabolite ratios imply lower formation and/or greater excretion of DM-6705 in pediatric subjects. Since DM-6705 contributes significantly to the QT-interval prolongation observed with delamanid, the lower ratios suggest that long-term delamanid dosing in children may result in lower QT prolongation compared to that in adults. Further information on this issue can be found in a comprehensive population pharmacokinetic analysis conducted in patients with pulmonary MDR-TB that explored the influence of age, bioavailability, and other factors on delamanid pharmacokinetics, as well as the relationship between plasma concentrations and corrected QT (QTc) interval (10).

The phase I and II studies provided the first detailed analysis of delamanid safety in children. Nearly all treatment-emergent adverse events in the 10-day phase I trial and 6-month phase II extension trial were mild to moderate in intensity; no patient permanently discontinued delamanid due to an adverse event, and no age-group-dependent trends in events were observed. The most common treatment-emergent adverse events were vomiting, pyrexia, nausea, toothache, and hyperuricemia in the phase I study and upper respiratory tract infection, headache, hyperuricemia, and arthralgia in the phase II study. Without a control group, it is difficult to attribute adverse events to delamanid or OBR, although many of the reported events are known to be related either to individual components of the tested combination regimen (e.g., hyperuricemia with pyrazinamide, arthralgia with pyrazinamide or levofloxacin, headache with delamanid) or to other common childhood illnesses (e.g., upper respiratory tract infection). Changes in ECG findings were within acceptable limits and in line with ECG and QT changes associated with adult delamanid use. Overall, this preliminary safety profile of delamanid in children tracks with the current safety profile in adults.

Conclusions regarding efficacy from this study should be considered provisional, given that the sample size was limited and that the study treatment (delamanid plus OBR) was not compared to an active control (e.g., OBR alone). With this caveat in mind, efficacy results were nonetheless encouraging: 89.1% of children had a favorable WHO-defined outcome (40.5% of children had achieved cure at 24 months and 48.6% had completed treatment). Furthermore, the new pediatric formulation was found to be highly palatable by child/parent self-reports and clinician observation in 0- to 5-year-olds.

In summary, the encouraging efficacy and safety profiles in this study, combined with the identification of a new child-friendly formulation, supports delamanid use in children 3 years of age and older with the formulations described in this study, consistent with WHO recommendations. Further evaluation regarding the most appropriate dose in children <3 years of age is a priority. Additional safety, efficacy, and pharmacokinetics (PK) analyses in younger children are under way in the following NIH-sponsored clinical trial: NCT03141060.

## MATERIALS AND METHODS

This report describes results from a phase I age deescalation study (ClinicalTrial.gov identifier: NCT01856634) and its phase II extension (NCT01859923), which assessed the safety, tolerability, pharmacokinetics, and efficacy of delamanid in children with MDR-TB who were receiving an OBR.

### Participants.

The phase I study enrolled children from birth to 17 years of age with confirmed or clinically diagnosed MDR-TB. A confirmed diagnosis required a positive culture and drug susceptibility test (DST) demonstrating resistance to isoniazid and rifampicin, or a positive genotypic DST demonstrating resistance to rifampicin alone or rifampicin and isoniazid from the child. A probable diagnosis required household or other close contact with a person with known MDR-TB or with a person who died while adherently receiving treatment for drug-susceptible TB, plus one of the following: a pathological specimen suggestive of TB, persistent cough lasting >2 weeks, fever, weight loss, and failure to thrive, or recent chest radiographs consistent with TB. A possible diagnosis was defined as probable TB disease in a child with contact of a source case with TB disease who either had risk factors for drug resistance or was being treated with first-line agents, with adequate adherence, without signs of clinical improvement (22). Children were excluded if they had Lansky play-performance scale scores of <50 (for children ≥1 year old) or Karnofsky performance status scale scores of <50, serious concomitant conditions, cardiac conditions, including clinically significant abnormalities in electrocardiogram (ECG) readings, and hepatitis B or C infection. Children living with HIV who were 1 to 5 years old or <1 year old were eligible if they had CD4 cell counts of ≥1,000/mm^3^ or ≥1,500/mm^3^, respectively.

The phase II extension enrolled children who had successfully completed the phase I study and remained <18 years at baseline.

### Study design.

**(i) Phase I study.** The first part of the study was a phase I, multicenter, open-label, uncontrolled, multiple-dose age deescalation trial. All children in the study received delamanid plus OBR per local standard of care for 10 days, followed by OBR alone for 8 days. The trial was conducted sequentially in four groups. Group 1 (12 to 17 years) and group 2 (6 to 11 years) received adult formulation delamanid (50-mg tablet) at 100 mg BID and 50 mg BID, respectively. Group 3 (3 to 5 years) received delamanid pediatric formulation (25 mg dispersible tablet) at a dose of 25 mg BID. Group 4 (birth to 2 years) received delamanid pediatric formulation (5 mg dispersible tablet) according to baseline body weight: >10 kg received 10 mg BID, >8 and ≤10 kg received 5 mg BID, and ≥5.5 and ≤8 kg received 5 mg once daily (QD). Doses used in this study were based on a population pharmacokinetic model of delamanid dosing in children (21) and the known pharmacokinetic and pharmacodynamic properties of delamanid in adults (18). Delamanid was administered with water and food in the morning and evening, approximately 10 h apart. Food composition was typical for age and country of origin. Following treatment, children were followed up at a safety visit 30 days after the last dose of delamanid.

**(ii) Phase II extension study.** The second part of the study was a phase II, open-label, multiple-dose age deescalation trial in children who had successfully completed the phase I study within the prior 30 days. Patients received 6 months of delamanid plus OBR therapy as described above for the phase I trial, followed by completion of treatment with OBR therapy alone. Final treatment outcome was assessed 1 year later, i.e., 24 months after the first dose of delamanid (18 months after the last dose of delamanid).

### Ethics.

The studies were conducted in the Philippines and South Africa from June 2013 to December 2017. Both were supported by Otsuka Pharmaceutical Development and Commercialization, Inc. (Rockville, MD, USA) and were in compliance with International Conference on Harmonization and Good Clinical Practice guidelines for conducting, recording, and reporting clinical trials. Written consent was obtained from each participant or his/her legal guardian (and written assent from children >7 years of age, as relevant). The informed consent form, protocol, and amendments for the study were approved by the institutional review board or independent ethics committee and by local health departments at each trial site.

### Assessments.

In the phase I study, pharmacokinetic blood draws occurred on days 1 to 2 and 10 to 11. Blood samples were drawn at 0 (predose), 2, 4, 10, 12 (groups 1 and 2 only), 14, and 24 h after the morning dose. Additional blood samples were drawn on days 13 (groups 1 and 2 only), 15, and 18 at 72, 120, and 192 h, respectively, after the last morning dose on day 10. Predose samples were taken within 45 min prior to dosing, and the 10-h sample was taken prior to the evening meal and evening dose of delamanid. Plasma concentrations of delamanid and its major metabolites were determined by high performance liquid chromatographic-tandem mass spectrometry (23). Pharmacokinetic parameters were determined by noncompartmental analysis. Sparse pharmacokinetic samples were also collected throughout the phase II study and will be utilized in a future population pharmacokinetic analysis.

Safety was assessed by treatment-emergent adverse event rate (including severity, seriousness, and relationship to study drug) and clinical laboratory results. ECGs were carried out during screening and on days −1, 1, 10, and 18 in the phase I study and during screening and on days −1, 1, 28, 56, 84, 126, 154, 182, and 210 in the phase II study. Each ECG assessment consisted of 3 consecutive 12-lead ECGs performed predose (if applicable) after the participant had been in a supine or semirecumbent position and at rest for ≥10 min. QT-interval length was corrected using Fridericia’s method (24).

Treatment response was assessed by sputum cultures, chest radiography (in children with pulmonary disease), body weight/height, and resolution of TB symptoms. Microbiological assessment of sputum or other biological specimens (e.g., smear microscopy, culture, identification, and DST) during follow-up was conducted at the discretion of the investigator as part of routine patient management. Palatability was assessed using an age-appropriate visual hedonic scale and by observer evaluation.

### Statistical analysis.

Outcomes were summarized for each age group and the entire population using descriptive statistics. No formal sample size calculation was performed.
