# Supplementary material for: Delamanid Added to an Optimized Background Regimen in Children with Multidrug-Resistant Tuberculosis: Results of a Phase I/II Clinical Trial
Source: Antimicrob Agents Chemother. 2022 Apr 11;66(5):e02144-21. doi: 10.1128/aac.02144-21 (PMC9112969; doi:10.1128/aac.02144-21)
Supplement: Supplemental file 1 — Fig. S1 and Tables S1 to S6. Download aac.02144-21-s0001.pdf, PDF file, 0.5 MB [file aac.02144-21-s0001.pdf]

Supplemental Data

Garcia-Prats AJ et al. *Delamanid Added to an Optimized Background Regimen in Children with Multidrug-Resistant Tuberculosis: Results of a Phase I/II Clinical Trial*

Supplement Figure 1. CONSORT Flow Diagram. Treated patients were those who received at least one dose of delamanid. Completed patients were those who were evaluated at the last scheduled visits of the individual trials (the day 18 follow-up visit in the phase I study and the 24-month follow-up visit in the phase II extension study).

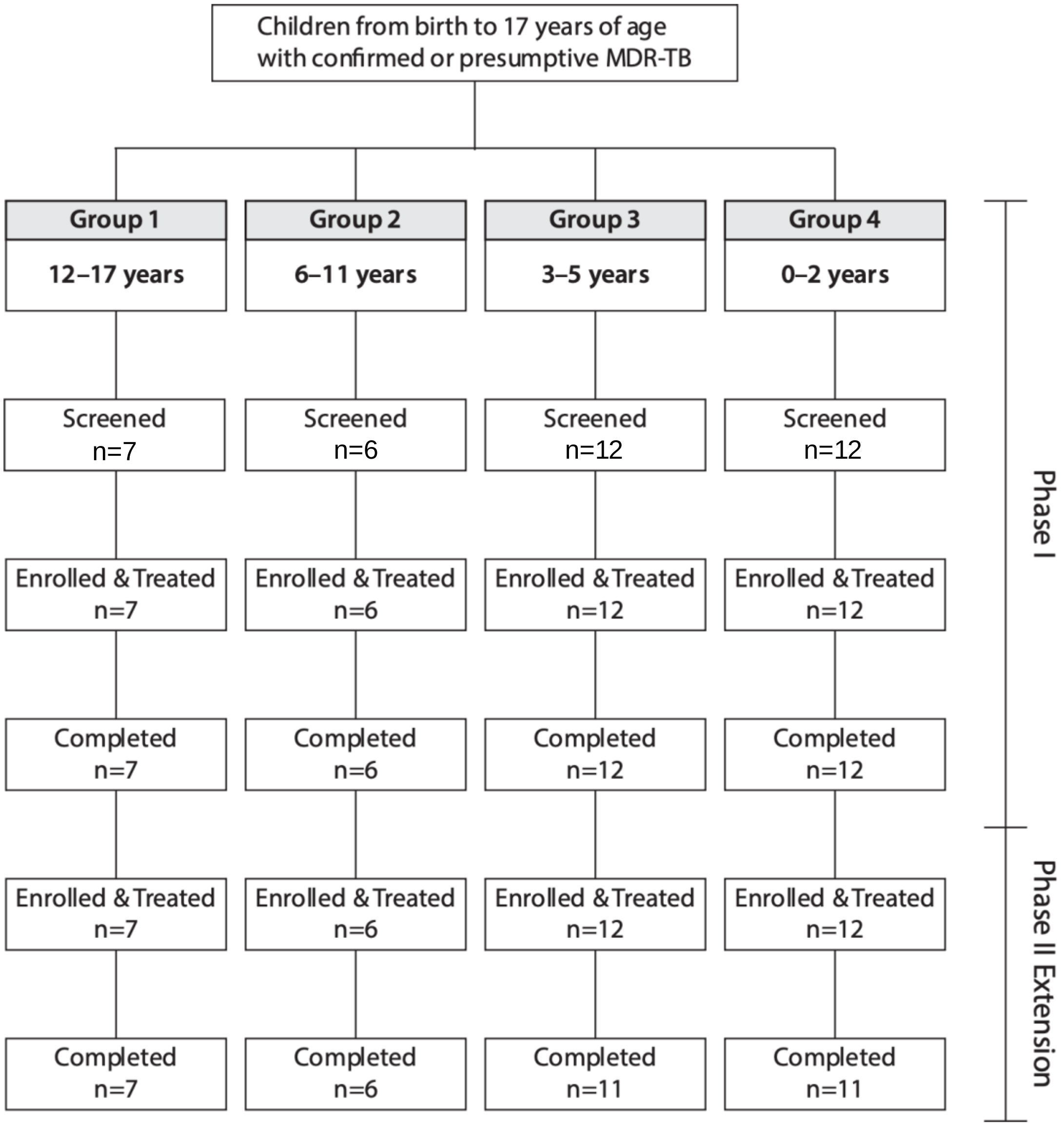

Supplement Table 1. Background Anti-TB Drugs by Age Group.

Anti-TB Agent, n (%)	12–17 years (N=7)	6–11 years (N=6)	3–5 years (N=12)	0–2 years (N=12)	Total (N=37)
Injectable aminoglycoside or capreomycin	7 (100%)	3 (50%)	9 (75%)	6 (50%)	25 (68%)
Levofloxacin	7 (100%)	6 (100%)	11 (92%)	12 (100%)	36 (97%)
Cycloserine or terizidone	7 (100%)	6 (100%)	12 (100%)	12 (100%)	37 (100%)
Prothionamide or ethionamide	1 (14%)	5 (83%)	10 (83%)	11 (92%)	27 (73%)
PZA	6 (86%)	4 (67%)	11 (92%)	11 (92%)	32 (86%)
Ethambutol	2 (29%)	2 (33%)	4 (33%)	6 (50%)	10 (27%)
PAS	6 (86%)	1 (17%)	4 (33%)	5 (42%)	16 (43%)
INH	0	2 (33%)	3 (25%)	6 (50%)	11 (30%)
Clofazimine	0	0	3 (25%)	6 (50%)	9 (24%)
Linezolid	0	0	1 (8%)	0	1 (3%)

Supplement Table 2. Pharmacokinetic Parameters for Delamanid and the Delamanid Metabolite DM-670-5 Following Oral Dosing on Day 1 in Children with Multidrug-Resistant Tuberculosis. Delamanid was administered in combination with an optimized background regimen. All values are expressed as median (range).

	**12–17 years (n=7)**	**6–11 years (n=6)**	**3–5 years (n=12)**	**0–2 years (n=12)** [a]
Delamanid dose	100mg BID	50mg BID	25mg BID	10mg BID<br>5mg BID,<br>or 5mg QD
Delamanid PK parameters				
C_{max} (ng/mL)	268<br>(164–420)	315<br>(205–454)	207<br>(150–364)	80.3<br>(26.2–121)
t_{max} (hr)	14·0<br>(2.05–24.0)	11.98<br>(2.0–24.0)	23.96<br>(14.0–24.03)	7.03<br>(2.0–24.0)
$AUC_{0–24hr}$ (ng×hr/mL)	3910<br>(1910–5270)	4080<br>(3240–7090)	3580<br>(1940–4920)	949<br>(262–1930)
DM-6705 PK parameter				
C_{max} (ng/mL)	8.60<br>(6.86–15.5)	7.68<br>(6.07–23.1)	8.35<br>(5.03–15.1)	2.01<br>(0.5–4.17)
t_{max} (hr)	24·0<br>(14.0–24.03)	18.98<br>(11·95–24.0)	23·98<br>(14.0–24.03)	24·0<br>(4.0–24.02)
$AUC_{0–24hr}$ (ng×hr/mL)	114<br>(89.4–224)	122<br>(81.1–351)	120<br>(77.9–223)	25.2<br>(2.49–61.8)

$AUC_{0–24hr}$, area under the plasma-time concentration curve from time 0 to 24 hours; BID, twice-daily; C_{max}, peak (maximum) concentration of drug in plasma; CL/F, oral clearance; ND, not determined; R_{ac}, accumulation ratio; $t_{½, z}$, terminal phase elimination half-life; t_{max}, time of peak concentration (C_{max}).

a. Group 4 received delamanid pediatric formulation according to their baseline body weights, i.e., patients who were >10 kg received 10mg BID, >8 and ≤10 kg received 5mg BID, and ≥5.5 kg and ≤8 kg received 5mg QD.

Supplement Table 3. Incidence of All Treatment-Emergent Adverse Events by System Organ Class and MedDRA Preferred Term in the Phase I Safety Population.

		12-17 YEARS (N=7) n[1] (%)		6-11 YEARS (N=6) n[1] (%)		3-5 YEARS (N=12) n[1] (%)		0-2 YEARS (N=12) n[1] (%)		TOTAL (N=37) n[1] (%)	
**SYSTEM ORGAN CLASS**	**ADVERSE EVENT (MEDDRA PREFERRED TERM)**	**n**	**(%)**	**n**	**(%)**	**n**	**(%)**	**n**	**(%)**	**n**	**(%)**
BLOOD AND LYMPHATIC SYSTEM DISORDERS	EOSINOPHILIA	0	(0.0)	0	(0.0)	1	(8.3)	1	(8.3)	2	(5.4)
	NEUTROPENIA	0	(0.0)	0	(0.0)	1	(8.3)	0	(0.0)	1	(2.7)
CARDIAC DISORDERS	CYANOSIS	1	(14.3)	0	(0.0)	0	(0.0)	0	(0.0)	1	(2.7)
EAR AND LABYRINTH DISORDERS	EAR PAIN	0	(0.0)	0	(0.0)	1	(8.3)	0	(0.0)	1	(2.7)
ENDOCRINE DISORDERS	HYPOTHYROIDISM	0	(0.0)	0	(0.0)	1	(8.3)	0	(0.0)	1	(2.7)
GASTROINTESTINAL DISORDERS	ABDOMINAL DISCOMFORT	0	(0.0)	1	(16.7)	0	(0.0)	0	(0.0)	1	(2.7)
	ABDOMINAL PAIN	2	(28.6)	0	(0.0)	0	(0.0)	0	(0.0)	2	(5.4)
	ABDOMINAL PAIN UPPER	0	(0.0)	0	(0.0)	1	(8.3)	0	(0.0)	1	(2.7)
	DIARRHEA	0	(0.0)	2	(33.3)	0	(0.0)	0	(0.0)	2	(5.4)
	FECES SOFT	1	(14.3)	0	(0.0)	0	(0.0)	0	(0.0)	1	(2.7)
	GASTRITIS	0	(0.0)	0	(0.0)	1	(8.3)	0	(0.0)	1	(2.7)
	GINGIVAL SWELLING	0	(0.0)	0	(0.0)	1	(8.3)	0	(0.0)	1	(2.7)
	LIP DRY	1	(14.3)	0	(0.0)	0	(0.0)	0	(0.0)	1	(2.7)
	MOUTH ULCERATION	0	(0.0)	0	(0.0)	2	(16.7)	0	(0.0)	2	(5.4)
	NAUSEA	4	(57.1)	0	(0.0)	1	(8.3)	0	(0.0)	5	(13.5)
	ORAL PAIN	1	(14.3)	0	(0.0)	0	(0.0)	0	(0.0)	1	(2.7)
	TOOTHACHE	1	(14.3)	2	(33.3)	2	(16.7)	0	(0.0)	5	(13.5)
	VOMITING	2	(28.6)	2	(33.3)	3	(25.0)	2	(16.7)	9	(24.3)
INJURY, POISONING AND PROCEDURAL COMP	CRANIOCEREBRAL INJURY	0	(0.0)	0	(0.0)	0	(0.0)	1	(8.3)	1	(2.7)
	EYE CONTUSION	0	(0.0)	0	(0.0)	0	(0.0)	1	(8.3)	1	(2.7)

INVESTIGATIONS	ELECTROCARDIOGRAM PR PROLONGATION	0	(0.0)	0	(0.0)	1	(8.3)	0	(0.0)	1	(2.7)
	ELECTROCARDIOGRAM QT PROLONGED	0	(0.0)	0	(0.0)	2	(16.7)	0	(0.0)	2	(5.4)
	ELECTROCARDIOGRAM U WAVE PRESENT	0	(0.0)	0	(0.0)	1	(8.3)	0	(0.0)	1	(2.7)
	PROTHROMBIN TIME PROLONGED	0	(0.0)	0	(0.0)	0	(0.0)	1	(8.3)	1	(2.7)
METABOLISM AND NUTRITION DISORDERS	DECREASED APPETITE	2	(28.6)	0	(0.0)	0	(0.0)	0	(0.0)	2	(5.4)
	HYPERURICEMIA	2	(28.6)	1	(16.7)	0	(0.0)	2	(16.7)	5	(13.5)
	HYPOKALEMIA	1	(14.3)	0	(0.0)	0	(0.0)	0	(0.0)	1	(2.7)
	HYPOMAGNESEMIA	1	(14.3)	0	(0.0)	0	(0.0)	0	(0.0)	1	(2.7)
MUSCULOSKELETAL AND CONNECTIVE TISSUE	ARTHRALGIA	2	(28.6)	1	(16.7)	1	(8.3)	0	(0.0)	4	(10.8)
	MUSCLE SPASMS	0	(0.0)	1	(16.7)	0	(0.0)	0	(0.0)	1	(2.7)
	MUSCULOSKELETAL PAIN	0	(0.0)	1	(16.7)	0	(0.0)	0	(0.0)	1	(2.7)
	PAIN IN EXTREMITY	1	(14.3)	0	(0.0)	0	(0.0)	. 0	(0.0)	1	(2.7)
	SOFT TISSUE SWELLING	0	(0.0)	1	(16.7)	0	(0.0)	0	(0.0)	1	(2.7)
NERVOUS SYSTEM DISORDERS	DIZZINESS	2	(28.6)	0	(0.0)	0	(0.0)	0	(0.0)	2	(5.4)
	HEADACHE	2	(28.6)	1	(16.7)	1	(8.3)	0	(0.0)	4	(10.8)
	PSYCHOMOTOR HYPERACTIVITY	0	(0.0)	0	(0.0)	1	(8.3)	0	(0.0)	1	(2.7)
PSYCHIATRIC DISORDERS	ABNORMAL BEHAVIOR	0	(0.0)	1	(16.7)	0	(0.0)	0	(0.0)	1	(2.7)
	HALLUCINATION	0	(0.0)	0	(0.0)	1	(8.3)	0	(0.0)	1	(2.7)
	INSOMNIA	1	(14.3)	0	(0.0)	0	(0.0)	0	(0.0)	1	(2.7)
RESPIRATORY, THORACIC AND MEDIASTINAL	BRONCHIAL HYPERREACTIVITY	0	(0.0)	0	(0.0)	0	(0.0)	3	(25.0)	3	(8.1)
	DYSPNEA	1	(14.3)	0	(0.0)	0	(0.0)	0	(0.0)	1	(2.7)
	HEMOPTYSIS	1	(14.3)	0	(0.0)	0	(0.0)	0	(0.0)	1	(2.7)
	OROPHARYNGEAL PAIN	0	(0.0)	1	(16.7)	0	(0.0)	0	(0.0)	1	(2.7)
SKIN AND SUBCUTANEOUS TISSUE DISORDER	BUTTERFLY RASH	0	(0.0)	1	(16.7)	0	(0.0)	0	(0.0)	1	(2.7)
	DERMATITIS DIAPER	0	(0.0)	0	(0.0)	0	(0.0)	3	(25.0)	3	(8.1)
	NIGHT SWEATS	1	(14.3)	0	(0.0)	0	(0.0)	0	(0.0)	1	(2.7)
	PRURITUS	2	(28.6)	0	(0.0)	0	(0.0)	0	(0.0)	2	(5.4)
	RASH MACULO-PAPULAR	0	(0.0)	0	(0.0)	0	(0.0)	1	(8.3)	1	(2.7)
	RASH PAPULAR	0	(0.0)	0	(0.0)	1	(8.3)	0	(0.0)	1	(2.7)
	SKIN HYPERPIGMENTATION	0	(0.0)	0	(0.0)	1	(8.3)	0	(0.0)	1	(2.7)
VASCULAR DISORDERS	HEMATOMA	0	(0.0)	0	(0.0)	1	(8.3)	0	(0.0)	1	(2.7)

**TOTAL[2]**		**5**	**(71.4)**	**5**	**(83.3)**	**9**	**(75.0)**	**12**	**(100.0)**	**31**	**(83.8)**

1. Subjects were counted once, per term, for the most severe of multiple occurrences of a specific MedDRA preferred term.
2. Subjects with adverse events in multiple System Organ Classes were counted only once toward the total.

Supplement Table 4. Incidence of All Treatment-Emergent Adverse Events by System Organ Class and MedDRA Preferred Term in the Phase II Safety Population.

	12–17 Years (N=7)	6–11 Years (N=6)	3–5 Years (N=12)	0–2 Years (N=12)	Total (N=37)
**System Organ Class**<br>**MedDRA Preferred Term**	**n (%)**	**n (%)**	**n (%)**	**n (%)**	**n (%)**
**At least one TEAE [a]**	7 (100.0)	6 (100.0)	12 (100.0)	12 (100.0)	37 (100.0)
**Blood and Lymphatic System Disorders**	2 (28.6)	0 (0.0)	1 (8.3)	1 (8.3)	4 (10.8)
Anemia	0 (0.0)	0 (0.0)	1 (8.3)	1 (8.3)	2 (5.4)
Eosinophilia	2 (28.6)	0 (0.0)	0 (0.0)	0 (0.0)	2 (5.4)
Immune Thrombocytopenic Purpura	0 (0.0)	0 (0.0)	0 (0.0)	1 (8.3)	1 (2.7)
**Cardiac Disorders**	1 (14.3)	0 (0.0)	0 (0.0)	0 (0.0)	1 (2.7)
Wolff-Parkinson-White Syndrome	1 (14.3)	0 (0.0)	0 (0.0)	0 (0.0)	1 (2.7)
**Ear and Labyrinth Disorders**	2 (28.6)	2 (33.3)	1 (8.3)	1 (8.3)	6 (16.2)
Cerumen Impaction	0 (0.0)	0 (0.0)	0 (0.0)	1 (8.3)	1 (2.7)
Conductive Deafness	0 (0.0)	1 (16.7)	0 (0.0)	0 (0.0)	1 (2.7)
Deafness Neurosensory	2 (28.6)	0 (0.0)	1 (8.3)	0 (0.0)	3 (8.1)
Middle Ear Effusion	0 (0.0)	1 (16.7)	0 (0.0)	0 (0.0)	1 (2.7)
**Endocrine Disorders**	0 (0.0)	1 (16.7)	2 (16.7)	2 (16.7)	5 (13.5)
Hypothyroidism	0 (0.0)	1 (16.7)	2 (16.7)	2 (16.7)	5 (13.5)
**Eye Disorders**	0 (0.0)	0 (0.0)	1 (8.3)	1 (8.3)	2 (5.4)
Conjunctivitis Allergic	0 (0.0)	0 (0.0)	0 (0.0)	1 (8.3)	1 (2.7)
Vernal Keratoconjunctivitis	0 (0.0)	0 (0.0)	1 (8.3)	0 (0.0)	1 (2.7)
**Gastrointestinal Disorders**	4 (57.1)	3 (50.0)	5 (41.7)	2 (16.7)	14 (37.8)
Abdominal Pain	1 (14.3)	1 (16.7)	1 (8.3)	0 (0.0)	3 (8.1)
Abdominal Pain Lower	1 (14.3)	0 (0.0)	0 (0.0)	0 (0.0)	1 (2.7)
Abdominal Pain Upper	0 (0.0)	0 (0.0)	1 (8.3)	0 (0.0)	1 (2.7)
Aphthous Ulcer	0 (0.0)	0 (0.0)	1 (8.3)	0 (0.0)	1 (2.7)
Constipation	0 (0.0)	0 (0.0)	1 (8.3)	0 (0.0)	1 (2.7)
Dental Caries	2 (28.6)	0 (0.0)	1 (8.3)	0 (0.0)	3 (8.1)
Dyspepsia	0 (0.0)	1 (16.7)	0 (0.0)	0 (0.0)	1 (2.7)
Gingival Swelling	1 (14.3)	0 (0.0)	0 (0.0)	0 (0.0)	1 (2.7)
Lip Dry	1 (14.3)	0 (0.0)	0 (0.0)	0 (0.0)	1 (2.7)
Lip Ulceration	0 (0.0)	0 (0.0)	0 (0.0)	1 (8.3)	1 (2.7)

	12–17 Years (N=7)	6–11 Years (N=6)	3–5 Years (N=12)	0–2 Years (N=12)	Total (N=37)
**System Organ Class<br>MedDRA Preferred Term**	**n (%)**	**n (%)**	**n (%)**	**n (%)**	**n (%)**
Mouth Ulceration	1 (14.3)	0 (0.0)	0 (0.0)	0 (0.0)	1 (2.7)
Oral Discomfort	1 (14.3)	0 (0.0)	0 (0.0)	0 (0.0)	1 (2.7)
Toothache	0 (0.0)	2 (33.3)	1 (8.3)	0 (0.0)	3 (8.1)
Vomiting	2 (28.6)	1 (16.7)	2 (16.7)	1 (8.3)	6 (16.2)
**General Disorders and Administration Site Conditions**	2 (28.6)	0 (0.0)	2 (16.7)	2 (16.7)	6 (16.2)
Infusion Site Extravasation	0 (0.0)	0 (0.0)	0 (0.0)	1 (8.3)	1 (2.7)
Pain	1 (14.3)	0 (0.0)	0 (0.0)	0 (0.0)	1 (2.7)
Pyrexia	2 (28.6)	0 (0.0)	2 (16.7)	1 (8.3)	5 (13.5)
**Infections and Infestations**	7 (100.0)	5 (83.3)	11 (91.7)	10 (83.3)	33 (89.2)
Acarodermatitis	0 (0.0)	0 (0.0)	0 (0.0)	3 (25.0)	3 (8.1)
Ascariasis	0 (0.0)	0 (0.0)	0 (0.0)	1 (8.3)	1 (2.7)
Bronchitis	1 (14.3)	0 (0.0)	1 (8.3)	0 (0.0)	2 (5.4)
Folliculitis	0 (0.0)	0 (0.0)	1 (8.3)	0 (0.0)	1 (2.7)
Gastroenteritis	1 (14.3)	1 (16.7)	1 (8.3)	4 (33.3)	7 (18.9)
Genital Candidiasis	0 (0.0)	0 (0.0)	1 (8.3)	0 (0.0)	1 (2.7)
Gingivitis	0 (0.0)	0 (0.0)	1 (8.3)	0 (0.0)	1 (2.7)
Helminthic Infection	0 (0.0)	0 (0.0)	1 (8.3)	0 (0.0)	1 (2.7)
Impetigo	0 (0.0)	0 (0.0)	0 (0.0)	1 (8.3)	1 (2.7)
Lower Respiratory Tract Infection	0 (0.0)	1 (16.7)	3 (25.0)	2 (16.7)	6 (16.2)
Mumps	0 (0.0)	0 (0.0)	0 (0.0)	1 (8.3)	1 (2.7)
Nasopharyngitis	3 (42.9)	0 (0.0)	0 (0.0)	0 (0.0)	3 (8.1)
Oral Candidiasis	1 (14.3)	0 (0.0)	1 (8.3)	0 (0.0)	2 (5.4)
Otitis Media	0 (0.0)	0 (0.0)	1 (8.3)	3 (25.0)	4 (10.8)
Otitis Media Acute	0 (0.0)	0 (0.0)	1 (8.3)	0 (0.0)	1 (2.7)
Parasitic Gastroenteritis	0 (0.0)	0 (0.0)	0 (0.0)	2 (16.7)	2 (5.4)
Pharyngotonsillitis	1 (14.3)	1 (16.7)	1 (8.3)	0 (0.0)	3 (8.1)
Pneumonia	1 (14.3)	1 (16.7)	3 (25.0)	2 (16.7)	7 (18.9)
Pustule	0 (0.0)	0 (0.0)	1 (8.3)	0 (0.0)	1 (2.7)
Pyelonephritis Acute	1 (14.3)	0 (0.0)	0 (0.0)	0 (0.0)	1 (2.7)
Pyuria	1 (14.3)	0 (0.0)	0 (0.0)	0 (0.0)	1 (2.7)
Respiratory Tract Infection	0 (0.0)	0 (0.0)	1 (8.3)	4 (33.3)	5 (13.5)

	12–17 Years (N=7)	6–11 Years (N=6)	3–5 Years (N=12)	0–2 Years (N=12)	Total (N=37)
**System Organ Class**<br>**MedDRA Preferred Term**	**n (%)**	**n (%)**	**n (%)**	**n (%)**	**n (%)**
Respiratory Tract Infection Viral	0 (0.0)	1 (16.7)	0 (0.0)	0 (0.0)	1 (2.7)
Rhinitis	1 (14.3)	2 (33.3)	0 (0.0)	0 (0.0)	3 (8.1)
Rubella	0 (0.0)	0 (0.0)	0 (0.0)	1 (8.3)	1 (2.7)
Subcutaneous Abscess	0 (0.0)	0 (0.0)	0 (0.0)	1 (8.3)	1 (2.7)
Systemic Viral Infection	0 (0.0)	0 (0.0)	1 (8.3)	0 (0.0)	1 (2.7)
Tinea Infection	0 (0.0)	0 (0.0)	0 (0.0)	1 (8.3)	1 (2.7)
Tinea Versicolor	0 (0.0)	0 (0.0)	0 (0.0)	1 (8.3)	1 (2.7)
Tooth Abscess	0 (0.0)	0 (0.0)	1 (8.3)	0 (0.0)	1 (2.7)
Upper Respiratory Tract Infection	4 (57.1)	3 (50.0)	5 (41.7)	2 (16.7)	14 (37.8)
Urinary Tract Infection	3 (42.9)	0 (0.0)	0 (0.0)	0 (0.0)	3 (8.1)
Viral Infection	1 (14.3)	1 (16.7)	0 (0.0)	0 (0.0)	2 (5.4)
Viral Upper Respiratory Tract Infection	1 (14.3)	1 (16.7)	0 (0.0)	0 (0.0)	2 (5.4)
Vulvovaginal Candidiasis	0 (0.0)	0 (0.0)	1 (8.3)	0 (0.0)	1 (2.7)
**Injury, Poisoning and Procedural Complications**	1 (14.3)	1 (16.7)	4 (33.3)	4 (33.3)	10 (27.0)
Animal Bite	1 (14.3)	0 (0.0)	0 (0.0)	0 (0.0)	1 (2.7)
Concussion	0 (0.0)	0 (0.0)	1 (8.3)	0 (0.0)	1 (2.7)
Contusion	0 (0.0)	0 (0.0)	1 (8.3)	0 (0.0)	1 (2.7)
Craniocerebral Injury	0 (0.0)	0 (0.0)	0 (0.0)	2 (16.7)	2 (5.4)
Eye Injury	0 (0.0)	0 (0.0)	0 (0.0)	1 (8.3)	1 (2.7)
Muscle Strain	1 (14.3)	0 (0.0)	0 (0.0)	0 (0.0)	1 (2.7)
Skin Abrasion	0 (0.0)	0 (0.0)	0 (0.0)	1 (8.3)	1 (2.7)
Skin Laceration	0 (0.0)	1 (16.7)	2 (16.7)	1 (8.3)	4 (10.8)
Tooth Injury	0 (0.0)	0 (0.0)	0 (0.0)	1 (8.3)	1 (2.7)
**Investigations**	1 (14.3)	0 (0.0)	4 (33.3)	6 (50.0)	11 (29.7)
Activated Partial Thromboplastin Time Prolonged	1 (14.3)	0 (0.0)	0 (0.0)	0 (0.0)	1 (2.7)
Alanine Aminotransferase Increased	0 (0.0)	0 (0.0)	1 (8.3)	0 (0.0)	1 (2.7)
Blood Corticotrophin Increased	0 (0.0)	0 (0.0)	2 (16.7)	1 (8.3)	3 (8.1)
Coagulation Time Prolonged	0 (0.0)	0 (0.0)	0 (0.0)	1 (8.3)	1 (2.7)
Hepatic Enzyme Increased	0 (0.0)	0 (0.0)	1 (8.3)	0 (0.0)	1 (2.7)

	12–17 Years (N=7)	6–11 Years (N=6)	3–5 Years (N=12)	0–2 Years (N=12)	Total (N=37)
**System Organ Class**<br>**MedDRA Preferred Term**	**n (%)**	**n (%)**	**n (%)**	**n (%)**	**n (%)**
Liver Function Test Increased	0 (0.0)	0 (0.0)	2 (16.7)	1 (8.3)	3 (8.1)
Prothrombin Time Prolonged	0 (0.0)	0 (0.0)	1 (8.3)	2 (16.7)	3 (8.1)
Weight Decreased	0 (0.0)	0 (0.0)	1 (8.3)	3 (25.0)	4 (10.8)
**Metabolism and Nutrition Disorders**	4 (57.1)	1 (16.7)	4 (33.3)	3 (25.0)	12 (32.4)
Decreased Appetite	1 (14.3)	0 (0.0)	0 (0.0)	0 (0.0)	1 (2.7)
Hyperuricemia	2 (28.6)	1 (16.7)	4 (33.3)	3 (25.0)	10 (27.0)
Hypokalemia	1 (14.3)	0 (0.0)	0 (0.0)	0 (0.0)	1 (2.7)
**Musculoskeletal and Connective Tissue Disorders**	5 (71.4)	2 (33.3)	4 (33.3)	2 (16.7)	13 (35.1)
Arthralgia	3 (42.9)	2 (33.3)	3 (25.0)	0 (0.0)	8 (21.6)
Arthritis	1 (14.3)	0 (0.0)	0 (0.0)	0 (0.0)	1 (2.7)
Bone Pain	1 (14.3)	0 (0.0)	0 (0.0)	0 (0.0)	1 (2.7)
Bursitis	1 (14.3)	0 (0.0)	0 (0.0)	0 (0.0)	1 (2.7)
Costochondritis	1 (14.3)	0 (0.0)	0 (0.0)	0 (0.0)	1 (2.7)
Muscular Weakness	0 (0.0)	0 (0.0)	0 (0.0)	1 (8.3)	1 (2.7)
Musculoskeletal Chest Pain	1 (14.3)	0 (0.0)	0 (0.0)	0 (0.0)	1 (2.7)
Myalgia	2 (28.6)	1 (16.7)	0 (0.0)	0 (0.0)	3 (8.1)
Pain In Extremity	1 (14.3)	0 (0.0)	0 (0.0)	1 (8.3)	2 (5.4)
Soft Tissue Swelling	0 (0.0)	0 (0.0)	1 (8.3)	0 (0.0)	1 (2.7)
**Neoplasms Benign, Malignant and Unspecified (Incl Cysts and Polyps)**	0 (0.0)	1 (16.7)	0 (0.0)	0 (0.0)	1 (2.7)
Non-Hodgkin's Lymphoma	0 (0.0)	1 (16.7)	0 (0.0)	0 (0.0)	1 (2.7)
**Nervous System Disorders**	5 (71.4)	3 (50.0)	4 (33.3)	1 (8.3)	13 (35.1)
Amnesia	0 (0.0)	0 (0.0)	1 (8.3)	0 (0.0)	1 (2.7)
Dizziness	2 (28.6)	0 (0.0)	0 (0.0)	0 (0.0)	2 (5.4)
Headache	5 (71.4)	3 (50.0)	2 (16.7)	0 (0.0)	10 (27.0)
Lethargy	0 (0.0)	0 (0.0)	0 (0.0)	1 (8.3)	1 (2.7)
Paresthesia	1 (14.3)	0 (0.0)	0 (0.0)	0 (0.0)	1 (2.7)
Psychomotor Hyperactivity	0 (0.0)	0 (0.0)	1 (8.3)	0 (0.0)	1 (2.7)
**Psychiatric Disorders**	1 (14.3)	1 (16.7)	1 (8.3)	1 (8.3)	4 (10.8)
Abnormal Behavior	0 (0.0)	1 (16.7)	0 (0.0)	0 (0.0)	1 (2.7)
Aggression	0 (0.0)	0 (0.0)	0 (0.0)	1 (8.3)	1 (2.7)

	12–17 Years (N=7)	6–11 Years (N=6)	3–5 Years (N=12)	0–2 Years (N=12)	Total (N=37)
**System Organ Class**<br>**MedDRA Preferred Term**	**n (%)**	**n (%)**	**n (%)**	**n (%)**	**n (%)**
Depression	1 (14.3)	0 (0.0)	0 (0.0)	0 (0.0)	1 (2.7)
Hallucination	0 (0.0)	0 (0.0)	1 (8.3)	0 (0.0)	1 (2.7)
Insomnia	0 (0.0)	1 (16.7)	0 (0.0)	0 (0.0)	1 (2.7)
**Renal and Urinary Disorders**	2 (28.6)	0 (0.0)	0 (0.0)	0 (0.0)	2 (5.4)
Dysuria	2 (28.6)	0 (0.0)	0 (0.0)	0 (0.0)	2 (5.4)
**Reproductive System and Breast Disorders**	1 (14.3)	0 (0.0)	0 (0.0)	0 (0.0)	1 (2.7)
Menorrhagia	1 (14.3)	0 (0.0)	0 (0.0)	0 (0.0)	1 (2.7)
**Respiratory, Thoracic and Mediastinal Disorders**	0 (0.0)	1 (16.7)	2 (16.7)	1 (8.3)	4 (10.8)
Asthma	0 (0.0)	0 (0.0)	1 (8.3)	0 (0.0)	1 (2.7)
Bronchial Hyperreactivity	0 (0.0)	0 (0.0)	0 (0.0)	1 (8.3)	1 (2.7)
Cough	0 (0.0)	0 (0.0)	1 (8.3)	0 (0.0)	1 (2.7)
Oropharyngeal Pain	0 (0.0)	1 (16.7)	0 (0.0)	0 (0.0)	1 (2.7)
**Skin and Subcutaneous Tissue Disorders**	1 (14.3)	2 (33.3)	2 (16.7)	5 (41.7)	10 (27.0)
Angioedema	0 (0.0)	0 (0.0)	1 (8.3)	0 (0.0)	1 (2.7)
Butterfly Rash	0 (0.0)	1 (16.7)	0 (0.0)	1 (8.3)	2 (5.4)
Dermatitis	0 (0.0)	0 (0.0)	0 (0.0)	1 (8.3)	1 (2.7)
Dermatitis Diaper	0 (0.0)	0 (0.0)	0 (0.0)	1 (8.3)	1 (2.7)
Eczema	0 (0.0)	0 (0.0)	0 (0.0)	1 (8.3)	1 (2.7)
Rash Maculo-Papular	1 (14.3)	0 (0.0)	0 (0.0)	0 (0.0)	1 (2.7)
Rash Papular	0 (0.0)	0 (0.0)	0 (0.0)	1 (8.3)	1 (2.7)
Skin Discoloration	0 (0.0)	0 (0.0)	0 (0.0)	1 (8.3)	1 (2.7)
Skin Fissures	1 (14.3)	0 (0.0)	0 (0.0)	0 (0.0)	1 (2.7)
Skin Hyperpigmentation	0 (0.0)	0 (0.0)	0 (0.0)	1 (8.3)	1 (2.7)
Skin Lesion	0 (0.0)	1 (16.7)	0 (0.0)	0 (0.0)	1 (2.7)
Urticaria	0 (0.0)	0 (0.0)	0 (0.0)	1 (8.3)	1 (2.7)
Urticaria Papular	0 (0.0)	0 (0.0)	1 (8.3)	0 (0.0)	1 (2.7)
**Social Circumstances**	0 (0.0)	0 (0.0)	1 (8.3)	0 (0.0)	1 (2.7)
Sexual Abuse	0 (0.0)	0 (0.0)	1 (8.3)	0 (0.0)	1 (2.7)

	12–17 Years (N=7)	6–11 Years (N=6)	3–5 Years (N=12)	0–2 Years (N=12)	Total (N=37)
**System Organ Class**<br>**MedDRA Preferred Term**	**n (%)**	**n (%)**	**n (%)**	**n (%)**	**n (%)**
**Vascular Disorders**	0 (0.0)	1 (16.7)	1 (8.3)	0 (0.0)	2 (5.4)
Hematoma	0 (0.0)	1 (16.7)	1 (8.3)	0 (0.0)	2 (5.4)

All adverse events which started after start of trial treatment; or if the event was continuous from baseline and was serious, study drug-related, or resulted in death, discontinuation, interruption, or reduction of trial treatment. Subjects were counted once, per term, for the most severe of multiple occurrences or a specific MedDRA preferred term.

a. Subjects with adverse events in multiple System Organ Classes were counted only once towards the total.

Supplement Table 5. Incidence of Electrocardiogram Abnormalities in the Phase I Safety Population. Baseline was defined as the average of the ECGs taken at Day 1.

Classification, n (%)	12–17 Years (N=7)	6–11 Years (N=6)	3–5 Years (N=12)	0–2 Years (N=12)	Total (N=37)
ECG population	7 (100.0)	6 (100.0)	12 (100.0)	12 (100.0)	37 (100.0)
Ventricular rate outliers					
Notable decreases [a]	0 (0.0)	0 (0.0)	0 (0.0)	0 (0.0)	0 (0.0)
Notable increases [b]	2 (28.5)	4 (66.6)	4 (33.3)	5 (41.6)	15 (40.5)
PR outliers [c]	0 (0.0)	0 (0.0)	0 (0.0)	0 (0.0)	0 (0.0)
QRS outliers [d]	0 (0.0)	0 (0.0)	0 (0.0)	0 (0.0)	0 (0.0)
QT >500 [e]	0 (0.0)	0 (0.0)	0 (0.0)	0 (0.0)	0 (0.0)
QTcB					
>500 [e] msec	0 (0.0)	0 (0.0)	0 (0.0)	0 (0.0)	0 (0.0)
>480 [e] msec	0 (0.0)	0 (0.0)	3 (25.0)	0 (0.0)	3 (8.1)
>450 [e] msec	2 (28.5)	2 (33.3)	8 (66.6)	3 (25.0)	15 (40.5)
Change ≥30 and ≤60 msec	2 (28.5)	1 (16.6)	9 (75.0)	7 (58.3)	19 (51.3)
Change >60 msec	0 (0.0)	0 (0.0)	1 (8.3)	0 (0.0)	1 (2.7)
QTcF					
>500 [e] msec	0 (0.0)	0 (0.0)	0 (0.0)	0 (0.0)	0 (0.0)
>480 [e] msec	0 (0.0)	0 (0.0)	0 (0.0)	0 (0.0)	0 (0.0)
>450 [e] msec	1 (14.2)	0 (0.0)	2 (16.6)	0 (0.0)	3 (8.1)
Change ≥30 and ≤60 msec	1 (14.2)	0 (0.0)	5 (41.6)	3 (25.0)	9 (24.3)
Change >60 msec	0 (0.0)	0 (0.0)	0 (0.0)	0 (0.0)	0 (0.0)
Abnormal U waves	0 (0.0)	0 (0.0)	1 (8.3)	0 (0.0)	1 (2.7)
ST segment changes	0 (0.0)	0 (0.0)	0 (0.0)	0 (0.0)	0 (0.0)
T wave changes	0 (0.0)	0 (0.0)	0 (0.0)	0 (0.0)	0 (0.0)

ECG, electrocardiogram; msec, milliseconds; QTcB, QTc interval corrected by Bazett's formula; QTcF, QTc interval corrected by Fridericia's formula.

a ≥25% decrease from baseline and ventricular rate <50 bpm.
b ≥25% increase from baseline and ventricular rate >100 bpm.
c ≥25% change from baseline when PR >200 msec.
d ≥25% change from baseline when QRS >100 msec.
e Patients who attained a value >450, >480 and >c 500 msec during the treatment period but not at each baseline.

Supplement Table 6. Incidence of Electrocardiogram Abnormalities in the Phase II Safety Population. Baseline was defined as the average of the ECGs taken at Day 1.

**Classification, n (%)**	**12–17 Years (N=7)**	**6–11 Years (N=6)**	**3–5 Years (N=12)**	**0–2 Years (N=12)**	**Total (N=37)**
ECG population	7 (100.0)	6 (100.0)	12 (100.0)	11 (91.7)	36 (97.3)
Ventricular rate outliers					
Notable decreases [a]	0 (0.0)	0 (0.0)	0 (0.0)	0 (0.0)	0 (0.0)
Notable increases [b]	1 (14.3)	0 (0.0)	1 (8.3)	4 (36.4)	6 (16.7)
PR outliers [c]	0 (0.0)	0 (0.0)	0 (0.0)	0 (0.0)	0 (0.0)
QRS outliers [d]	1 (14.3)	0 (0.0)	0 (0.0)	0 (0.0)	1 (2.8)
QT >500 [e] msec	0 (0.0)	0 (0.0)	0 (0.0)	0 (0.0)	0 (0.0)
QTcB					
>500 [e] msec	0 (0.0)	0 (0.0)	0 (0.0)	0 (0.0)	0 (0.0)
>480 [e] msec	1 (14.3)	1 (16.7)	1 (8.3)	0 (0.0)	3 (8.3)
>450 [e] msec	7 (100.0)	2 (33.3)	8 (66.7)	5 (45.5)	22 (61.1)
Change ≥30 and ≤60 msec	5 (71.4)	3 (50.0)	8 (66.7)	9 (81.8)	25 (69.4)
Change >60 msec	1 (14.3)	0 (0.0)	0 (0.0)	2 (18.2)	3 (8.3)
QTcF					
>500 [e] msec	0 (0.0)	0 (0.0)	0 (0.0)	0 (0.0)	0 (0.0)
>480 [e] msec	0 (0.0)	0 (0.0)	0 (0.0)	0 (0.0)	0 (0.0)
>450 [e] msec	3 (42.9)	2 (33.3)	0 (0.0)	0 (0.0)	5 (13.9)
Change ≥30 and ≤60 msec	5 (71.4)	2 (33.3)	6 (50.0)	9 (81.8)	22 (61.1)
Change >60 msec	1 (14.3)	0 (0.0)	0 (0.0)	1 (9.1)	2 (5.6)
Abnormal U waves	0 (0.0)	0 (0.0)	0 (0.0)	0 (0.0)	0 (0.0)
ST segment changes	0 (0.0)	0 (0.0)	0 (0.0)	0 (0.0)	0 (0.0)
T wave changes	0 (0.0)	0 (0.0)	0 (0.0)	0 (0.0)	0 (0.0)

ECG, electrocardiogram; msec, milliseconds; QTcB, QTc interval corrected by Bazett's formula; QTcF, QTc interval corrected by Fridericia's formula.

a ≥25% decrease from baseline and ventricular rate <50 bpm.
b ≥25% increase from baseline and ventricular rate >100 bpm.
c ≥25% change from baseline when PR >200 msec.
d ≥25% change from baseline when QRS >100 msec.
e Patients who attained a value >450, >480 and >500 msec during the treatment period but not at each baseline.
